# The emerging roles of NGS-based liquid biopsy in non-small cell lung cancer

**DOI:** 10.1186/s13045-017-0536-6

**Published:** 2017-10-23

**Authors:** Yi-Chen Zhang, Qing Zhou, Yi-Long Wu

**Affiliations:** Guangdong Lung Cancer Institute, Guangdong General Hospital, Guangdong Academy of Medical Sciences, 106 Zhongshan 2nd Road, Guangzhou, Guangdong 510080 People’s Republic of China

**Keywords:** Next-generation sequencing, Liquid biopsy, ctDNA

## Abstract

The treatment paradigm of non-small cell lung cancer (NSCLC) has evolved into oncogene-directed precision medicine. Identifying actionable genomic alterations is the initial step towards precision medicine. An important scientific progress in molecular profiling of NSCLC over the past decade is the shift from the traditional piecemeal fashion to massively parallel sequencing with the use of next-generation sequencing (NGS). Another technical advance is the development of liquid biopsy with great potential in providing a dynamic and comprehensive genomic profiling of NSCLC in a minimally invasive manner. The integration of NGS with liquid biopsy has been demonstrated to play emerging roles in genomic profiling of NSCLC by increasing evidences. This review summarized the potential applications of NGS-based liquid biopsy in the diagnosis and treatment of NSCLC including identifying actionable genomic alterations, tracking spatiotemporal tumor evolution, dynamically monitoring response and resistance to targeted therapies, and diagnostic value in early-stage NSCLC, and discussed emerging challenges to overcome in order to facilitate clinical translation in future.

## Background

In 1976, the relationship between genetic instability and tumorigenesis was proposed by Nowell [1]. Later on, progress in cancer genomics has further strengthened the notion that cancer is driven by various types of genomic alterations [2]. Global advances in sequencing techniques have refined an evolving genomic landscape of cancer. The technical revolution began with the era of first-generation sequencing, in which the Human Genome Project was an outstanding landmark by depicting the first 99.7% complete human genome with about 22,000 genes involved [3–5]. With the use of capillary-based instrument, methodologies of first-generation sequencing offered important discoveries of candidate genes [6–8], meanwhile raising concerns on cost-effectiveness and limitations in the scope of sequencing. Consequently, the sequencing pattern gradually developed into genomic scale fashion during the second half of last decade. Starting in 2005, next-generation sequencing (NGS) enabled a comprehensive profiling of cancer genome with unprecedented depth and breadth [2]. Collaborative projects such as The Cancer Genome Atlas (TCGA) and the International Cancer Genome Consortium (ICGC) have characterized pan-cancer genetic abnormalities using NGS, cumulating knowledge of cancer genomics to accelerate discoveries of cancer causes and to improve diagnosis and treatment [9, 10]. Besides, emerging technical advances have made the cost of NGS to dramatically decrease and reach the point where an entire human genome could be sequenced for less than $1000 [11], increasing its accessibility to researchers.

NGS has three major advantages over the first-generation Sanger sequencing. The most outstanding one is its high-throughput, making testing of thousands of genes or even the whole genome possible. Additionally, NGS demonstrates excellent testing performance with compatibility of low-input DNA. Lastly, NGS appears to be more cost-effective in massively parallel sequencing [12, 13]. These abovementioned advantages have made NGS a promising testing for NSCLC, where multiple agents targeting various actionable genomic alterations are available [14–22]. Meanwhile, challenges of regulatory issues, assay validation, proficiency testing, and quality control still need to be overcome [23]. With more and more NGS platforms developed, standardization of reports from various test platforms is also urgently warranted.

Liquid biopsy is another revolutionary advance in genomic profiling of NSCLC. Currently, there are three types of circulating biomarkers that can be detected in liquid biopsy: circulating tumor DNA (ctDNA), circulating tumor cells (CTCs), and exosomes [24]. Among them, ctDNA is a potential surrogate for the entire tumor genome, and it is often referred to as “liquid biopsy” [25]. Liquid biopsy has advantages over traditional tissue biopsy in that the procedure is minimally invasive, is able to reflect a comprehensive genome landscape contributed by multiple tumor sites, and has the potential in serial monitoring [26]. Liquid biopsy demonstrated promising reference value in diagnosis and treatment of advanced NSCLC. Meanwhile, issues remain, including varying sensitivities and specificities between different platforms and lack of standardization of techniques and downstream processing [27].

**Fig. 1:**
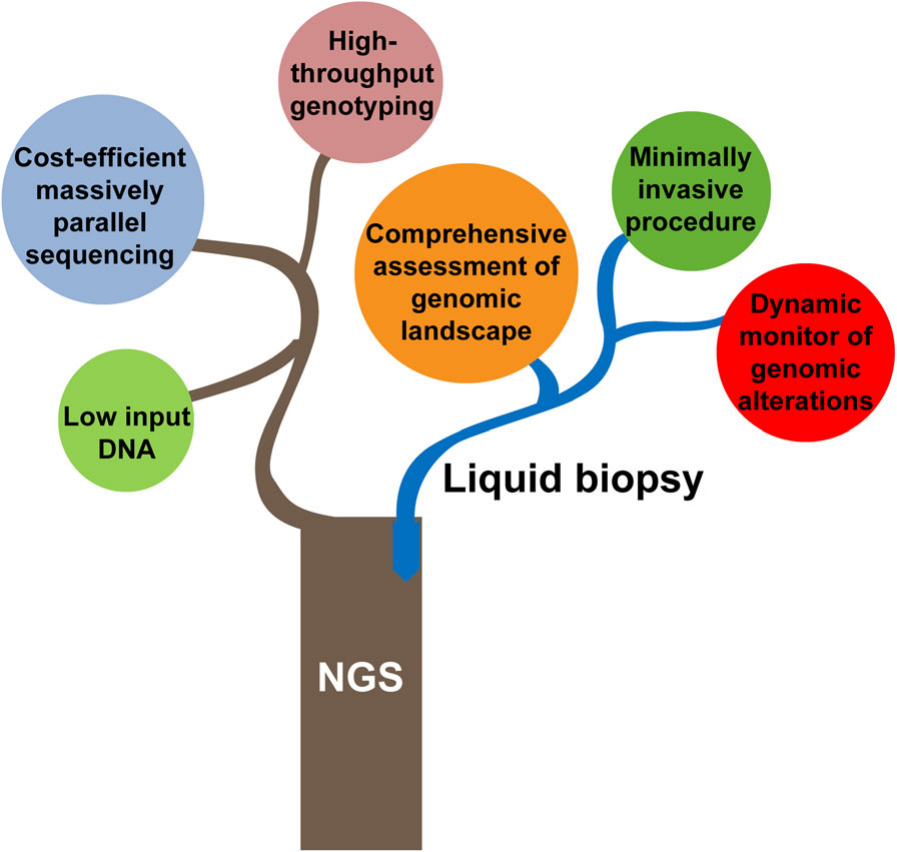
Integration of NGS and liquid biopsy maximizes overall advantages

The integration of NGS with liquid biopsy seems like the grafting in agriculture, which maximizes overall advantages (Fig. 1). NGS-based liquid biopsy might facilitate a minimally invasive and comprehensive genomic profiling of NSCLC that overcomes spatial heterogeneity arising from tissue biopsy and limitations in genomic information from candidate gene characterization. There is an increasing number of studies demonstrating the utility of NGS-based liquid biopsy in both advanced and early-stage NSCLC. Recent large-scale genomic profiling of advanced NSCLC by NGS-based ctDNA assays have demonstrated high concordance with matched tissue [28, 29], but it is noteworthy that the accuracy of this test in resectable stage NSCLC have reported to be much lower ranging from 23.3 to 50.4% [30, 31], possibly due to the low concentrations of ctDNA in early-stage patients. Along with efforts in improving the test sensitivity and investigations in diagnosis and monitoring NSCLC come practical challenges. This review summarizes the applications and emerging challenges of NGS-based liquid biopsy in patients with NSCLC.

## Identifying actionable genomic alterations in patients with advanced NSCLC

The National Comprehensive Cancer Network guideline of NSCLC recommended testing for seven biomarkers amenable to targeted therapies, including epidermal growth factor receptor (EGFR) mutation, fusions in anaplastic lymphoma kinase (ALK), c-ros oncogene 1 receptor (ROS1) and RET proto-oncogene (RET), mesenchymal–epithelial transition (MET) amplification or MET exon 14 skipping mutation, human epidermal growth factor receptor-2 (HER2) mutation, and BRAF V600E mutation, indicating the necessity of multiplex sequencing. NGS-based ctDNA assay has demonstrated the feasibility of identifying multiple actionable genomic alterations with overall concordance rates with matched tissue ranging from 60 to 86% across various platforms in patients with advanced lung cancer [32–37]. A bias-corrected targeted NGS detected a broad range of actionable genomic alterations in the plasma, including *ALK*, *ROS1*, and *RET* rearrangements, *HER2* insertions, and *MET* amplification in advanced NSCLC, with 100% specificity [34]. Another study using a semi-conductor-based NGS platform identified multiple biomarkers in plasma ctDNA including *EGFR*, *KRAS*, *PIK3CA*, and *TP53* with an overall concordance rate of 76% with paired tissue DNA [32]. A proof-of-concept study from BioCAST/IFCT-1002 also reported the utility of NGS-based ctDNA assay to screen clinically relevant biomarkers including *EGFR*, *KRAS*, *BRAF*, *ERBB2*, and *PI3KCA* with an overall sensitivity of 58% and estimated specificity of 86% [33]. Notably, NGS-based ctDNA assay has demonstrated impressive performance of genotyping in cases of incomplete or negative tissue genotyping [29, 33, 34, 38–40]. In a recent study evaluating the utility of ctDNA analysis by digital NGS of over 8000 advanced NSCLC, additional actionable biomarkers such as *EGFR* mutations, *ALK* and *ROS* fusion, *BRAF* V600E mutation, and *Met* 14 skipping mutation were identified in 29% of unvaluable or under genotyped tissue cases [29]. Additionally, these evidences also suggested that NGS-based ctDNA assay might appear as a cost-effective approach to offer patients with advanced NSCLC more opportunities to be enrolled in innovative clinical studies involving multiple biomarkers analysis such as umbrella and cluster trials.

Among the abovementioned actionable genomic alterations, the performance of NGS in discovering druggable mutations is of clinical significance. Regarding EGFR testing, NGS-based ctDNA assay showed preferable sensitivity and specificity in detecting *EGFR* exon 19 deletion, exon 21 L858R mutation, and exon 20 T790M mutation. In TIGER-X study, a short footprint mutation enrichment NGS platform was used to interrogate EGFR activating mutations and T790M mutation in the urine and plasma samples from patients [39]. With tissue as a reference, the sensitivity of EGFR mutation detection in plasma was 87, 100, and 93% for exon 19 deletion, exon 21 L858R mutation, and exon 20 T790M mutation, respectively. The specificity of plasma EGFR mutation detection was 96% for exon 19 deletion, 100% for exon 21 L858R mutation, and 94% for exon 20 T790M mutation. The sensitivity of urine EGFR mutation detection in specimens that met the recommended volume of 90–100 ml also reached 83, 80, and 93% for exon 19 deletion, exon 21 L858R, and exon 20 T790M mutation, respectively. In a prospective study enrolling 288 NSCLC patients, the diagnostic specificity of NGS for exon 19 deletions and exon 21L858R mutation in the plasma were 98 and 94.1%, respectively, indicating a positive ctDNA result might enable direct recommendation of EGFR TKIs. The overall testing sensitivity was 72.7% in stage IIIB–IV patients [41]. Another issue that deserves to be addressed is the clinical accuracy of plasma EGFR assays as compared to matched tissue biopsies. Evidences from current largest data enrolling 229 advanced NSCLC patients with matched NGS-based ctDNA and tissue tests demonstrated that the positive predictive value (PPV) of ctDNA sequencing was 100% for *EGFR* exon 21 L858R mutation, 98% for *EGFR* exon 19 deletion, and 27% for *EGFR* exon 20 T790M mutation, suggesting latter acquisition of this resistance mutation [42]. The difference in plasma test accuracy between *EGFR* T790M mutation and *EGFR*-activating mutations was also reported in AURA3 study. Paired cobas plasma and tissue tests also demonstrated that the positive percent agreement of cobas plasma test with the cobas tissue test for EGFR T790M mutation detection was 51%, lower than that for EGFR activating mutations (exon 19 deletion, 82%; exon 21 L858R mutation, 68%) [43]. The abovementioned data highlighted the necessity of routine tissue biopsy in cases of EGFR T790M mutation-negative plasma assay.

Besides, the utility of NGS in plasma ALK testing was also demonstrated in several studies [34, 36, 38, 44]. Comparing two studies which performed non-invasive genotyping of ALK fusion by capture-based NGS, the test accuracy ranged from 68.8 to 91.7% for advanced NSCLC [38, 44]. Additionally, rare ALK fusion types, such as FAM179A-ALK and COL25A1-ALK, and ALK mutations including ALK L1152R, ALK I1171T, and ALK L1196M were also identified. With the advantages of simultaneous screening rare or even unknown ALK fusion patterns as well as somatic mutations, NGS-based ctDNA assay might not only provide a more comprehensive landscape of advanced ALK-positive NSCLC, but also offer more opportunities of ALK inhibitors to this subset of patients.

Of these druggable mutations, one important issue is whether the NGS ctDNA testing is comparable or even superior to routine methodologies. Evidence from multiple studies of EGFR mutation detection has demonstrated that the testing performance of NGS platforms is relatively comparable to polymerase chain reaction (PCR)-based and cobas platforms [45, 46]. Direct comparison was reported in AURA II study where cobas (a FDA-approved plasma EGFR test) and NGS were involved in plasma EGFR T790M testing. As compared to MiSeq NGS, the sensitivity and specificity of cobas was 91.5 and 91.1%, respectively. The concordance rate between the two methods was 91.3% [45]. Such performance was also comparable to that of BEAMING and digital droplet PCR (ddPCR) as reported in AURA I study. As compared to ARMS, the sensitivity and specificity ranged from 22.1 to 75% and 85 to 100%, respectively, in various studies [47]. NGS seemed to exhibit equivalent or even superior performance in plasma EGFR testing.

The biggest challenge facing NGS-based liquid biopsy is whether discoveries are really actionable in clinical practice. In case that multiple actionable genomic alterations are identified, how to distinguish the driver genes to be targeted from other passengers? Technically, this question might not be easily solved. With the background rates of mutation greatly vary among different patients and regions of the genome, NGS could not reliably indicate the driver genes from a number of passengers [48]. Besides, there is no universal consensus on the valid cut-off point of these actionable mutations being clinically relevant to take action. Several studies have reported cases with ultra-low mutation frequency in ctDNA [29, 33, 35]. NGS-based ctDNA assay of patients with IIIb/IV NSCLC showed that 46% of somatic mutations detected in ctDNA were observed at a frequency below 1% [35]. Whether targeting these actionable alterations with ultra-low frequency is clinically meaningful remains unknown. Thus, future studies deserve to further distinguish driver genetic alterations and define the valid cut-off point with clinical significance to make discoveries from NGS-based ctDNA assay becoming actionable in clinical practice. The APPLE Trial (NCT02856893), a randomized, three-arm, phase II study evaluating the feasibility and activity of osimertinib treatment on positive plasma EGFR T790M mutation in EGFR-mutant NSCLC patients might hopefully provide further evidence on this issue [49].

Discrepancies between liquid and tissue biopsy seem to be another challenge that hinders clinical translation. Both tissue-positive ctDNA-negative, and ctDNA-positive tissue-negative cases have been reported in several studies [33, 34, 38, 39]. Therefore, NGS-based liquid biopsy appears to complement the gold standard tissue biopsy. Integrating tissue testing and NGS-based liquid biopsy might be a promising strategy in molecular profiling of advanced NSCLC in the future. Meanwhile, due to the differences in NGS platforms and tissue assays across published studies, further evidences are needed to define the relationship of NGS-based ctDNA assays and tissue tests to facilitate clinical translation. Results from an ongoing observational study (NCT02620527) which compares the concordance between ctDNA assay and matched tissue test by FoundationOne is worth the expectation [50].

## Dynamic monitoring of response and resistance to targeted therapies

Firstly, the value of NGS-based ctDNA analysis in predicting responses to targeted therapies has been demonstrated. Quantification of circulating specific tumor genes by NGS has been shown to be potential surrogate markers for patients under targeted treatments. Several studies suggested that the extent to which plasma and urine EGFR mutation levels drop after initiation of EGFR TKI might predict depth of response [39, 51, 52]. Of note, the ctDNA responses correlated well with radiologic responses in radiologic good responders, whereas correlation was poor in non-responders to EGFR TKIs [53]. In addition to quantification of specific tumor genes, the changes in molecular tumor load (MTL) detected by NGS correlated with or predicted all (95% CI, 82.0–99.8%) radiological and/or clinical responses except for cases without any genomic alteration detected [54].

Secondly, NGS-based ctDNA assay has also been reported to provide prognostic implications for patients with advanced NSCLC. ctDNA positive at diagnosis was suggested to be an independent marker of poor prognosis, with a median overall survival (OS) of 13.6 months versus 21.5 months (adjusted hazard ratio [HR] 1.82, *p* = 0.045). In addition, ctDNA clearance at first evaluation was also correlated with OS independently of Response Evaluation Criteria in Solid Tumors (RECIST) (HR 3.27, *p* < 0.001) [55]. Similarly, another prospective study further found that a cell-free DNA (cfDNA) concentration > 3 ng/l was associated with a decreased OS (median, 24 vs. 46 months; log-rank, *p* < 0.01) [36].

Thirdly, NGS-based ctDNA assay has also been successfully used for dynamically monitoring actionable genomic alterations [56–58]. Plasma EGFR monitoring by deep sequencing demonstrated that the mutation detection rate of EGFR exon 19 deletion/exon 21 L858R mutation was high at the initiation of EGFR-TKI (*p* = 0.001), suppressed during treatment course before disease progression, and elevated after the onset of disease progression (*p* = 0.023). The mutation detection rate of EGFR T790M was low until the onset of disease progression and elevated thereafter (*p* = 0.01) [56]. Another example of the ability of NGS-based ctDNA assay to dynamically monitor actionable genomic alternations was demonstrated in an ALK-positive case. The MAF of ALK fusion was detected at 0.91% pre-treatment and dropped to 0.41% at progression along with the emergence of F1174C which was detected at 1.0% [58].

Lastly, NGS-based ctDNA assay has refined a more heterogeneous resistance landscape to targeted therapies. Plasma NGS at pretreatment of rociletinib demonstrated a much more heterogeneous resistance scenario to first-line EGFR TKIs [59]. Concurrent with EGFR T790M mutation, multiple additional resistance mechanisms were observed in 46% of patients after failure from prior EGFR inhibitors, much higher than 5–15% in previous reports [60–63]. NGS-based ctDNA assay has also discovered novel resistance mechanisms to third-generation EGFR inhibitors. A well-known example is the discovery of EGFR C797S mutation, which mediates to osimertinib [64–67]. Intriguingly, NGS ctDNA analysis not only identified EGFR C797S mutation at disease progression, but also further demonstrated different genomic presentations of acquired EGFR C797S mutation, in cis or in trans with EGFR T790M mutation. In addition to the same DNA alteration seen in the tumor samples, plasma ctDNA analysis identified a second DNA alteration encoding the C797S mutation. Other novel resistance mechanisms to third-generation EGFR inhibitors such EGFR C797G mutation and EGFR L798I mutation have also been identified by NGS of pleural effusion and ctDNA, respectively [59, 68]. NGS-based ctDNA assay also unveiled a more comprehensive resistance landscape of ALK inhibitor crizotinib including F1174C/V, G1202R, L1198F, I1171T, and L1196M and potential novel resistance mechanisms of co-occurring SNVs in other genes that are absent in treatment-naïve patients [58].

The major hurdle in monitoring resistances is to identify dominant resistance mechanisms to guide what clinical action to be taken. Similar to tissue-based NGS genomic profiling, on condition that NGS-based ctDNA assay demonstrates a resistant landscape with two or more actionable genomic alterations, how should clinicians take action? One potential approach is to systematically use cellular characterization, clonal analysis, and protein structure to further validate discoveries from NGS-based ctDNA assay and moreover to suggest potential targeted agents. The feasibility of similar strategy has been demonstrated in a case report of advanced ALK-positive NSCLC. Systematic use of multiple assays not only validated that ALK C1156Y-L1198F mutations induced resistance to lorlatinib but also suggested the regained sensitivity to crizotinib, providing evidence on recalling crizotinib as a subsequent treatment to overcome lorlatinib resistance [69].

Another emerging challenge is how to refine treatment strategy according to ctDNA mutational dynamics. An innovative strategy has been proposed in a trial which plans to monitor tumor resistance by ctDNA and tailor treatment based on abundance of EGFR T790M mutation in plasma [70]. When the levels of EGFR T790M mutation reduce, they will switch to a first-generation EGFR inhibitor, and when EGFR T790M mutation levels rise, the researchers will switch back to osimertinib. Collectively, endeavors to investigate novel treatment strategies based on ctDNA dynamics is promising; however, present evidence are not yet sufficient to transform clinical practice.

## Diagnostic value in early-stage NSCLC

The potential of NGS-based ctDNA in screening for early-stage NSCLC has been demonstrated in several studies. The median yield of cfDNAs was demonstrated to be significantly higher in patients with early-stage lung adenocarcinomas, as compared to healthy controls (4.9 vs. 2.32 ng, *p* = 0.003). In addition, Log2 ratio-based CNV analysis demonstrated subtle but detectable differences in cfDNAs between patients and controls, suggesting that such assay may sensitively distinguish early-stage disease when in combination with other existing screening strategies such as low-dose CT scanning [71]. In addition, the feasibility of detecting genomic alterations in ctDNA/cfDNA by NGS for early-stage NSCLC have been reported. A prospective study conducted in surgical stage I NSCLC showed that the overall concordance rate between tDNA and matched NGS-based ctDNA assay was 50.4%, with a sensitivity of 53.8%, a specificity of 47.3%, and a plasma PPV of 53.2% [31]. As the test sensitivity is a key point in detecting early-stage NSCLC with low levels of ctDNA/cfDNA, interpreting these results has to be careful and further evaluation are warranted. In addition to identifying early-stage NSCLC, the ability of detecting both ubiquitous and heterogeneous SNVs by NGS-based cfDNA assay has also been reported in a pilot study, revealing intratumor heterogeneity in early-stage NSCLC [72]. Moreover, a promising strategy that enabled early detection and mapping the primary growth site of a tumor was recently demonstrated by characterization of methylation haplotyping in plasma cfDNA via the combined use of whole-genome bisulfite sequencing and other analysis in 59 patients with lung or colorectal cancer [73].

The use of NGS-based liquid biopsy in screening for early-stage NSCLC is challenging, as an increased risk of false-positive results is more likely with the increased sensitivity [74]. Clinical concerns on early-stage cancer detection by ctDNA have arisen [75], whether oncologists should take action or follow “watch and wait” strategy in case of positive screening results. Integrating the use of low-dose CT screening with NGS-based liquid biopsy might hopefully reduce the lead-time bias and facilitate early detection in high-risk population for lung cancer. However, how shall clinicians take action in case one has positive findings from NGS-based liquid biopsy but no imaging abnormalities remains a challenge to be solved in the future.

## Tracking spatiotemporal tumor evolution

With the application of NGS, intratumoral clonal heterogeneity has been demonstrated to be a key factor fostering therapeutic resistance to anti-cancer treatments [76]. Spatiotemporal tumor evolution undertreatment selection might be the root of intratumoral heterogeneity. NGS-based liquid biopsy has appeared to be a valuable approach to decipher the spatiotemporal tumor evolution of lung cancer. Two evolving patterns of MTL have been identified in a recent study which performed serial monitor of ctDNA from 38 patients with advanced lung cancer (NSCLC accounting for 95%) by digital NGS [54]. One pattern is the clonal changes while receiving targeted therapy; the other is the global changes to PD-1 checkpoint inhibitors, chemotherapy, or radiation. Additionally, a large observational study called Tracking Lung Cancer Evolution Through Treatment (TRACERx) has been launched (NCT01888601) to depict the spatiotemporal evolution trace of early-stage lung cancer [77]. A bespoke multiplex-PCR NGS approach to ctDNA profiling in the first 100 TRACERx has demonstrated the feasibility to characterizing the subclonal dynamics of relapsing NSCLC and identifying the emerging subclones prior to clinical recurrence. By following both clonal and subclonal single nucleotide variants (SNVs) present in pre-operative plasma and at the time of recurrence, the study found that 48% of patients had ≥ 2 detectable SNVs in ctDNA. The median interval between ctDNA detection and relapse detected by CT scanning was 70 days. Besides, the phylogenetic origin of the metastatic subclone could be traced [78]. Just as the Chinese strategist Sun Tzu ever said, “Know yourself and know your enemy, you could win every war”. NGS-based liquid biopsy might potentially contribute to piecing together a more precise picture of how tumor evolve and help us evolve our treatment strategies accordingly to reshape this heterogeneous population.

Along with deeper insights into lung cancer come novel challenges. As mentioned in the study of ctDNA profiling in the first 100 TRACERx, the estimated cost per patient for sequencing of a single tumor region, synthesis of a patient-specific assay panel, and profiling of five plasma sample is $1750. How to incorporate novel findings from these high-cost studies into improving clinical outcomes is a key practical question. One potential approach might be adapting treatment strategy according to evolutionary dynamics to improve the efficacy of current available agents. DARWIN I study (NCT02183883), which involves patients registered to TRACERx study, will assess if targeting *EGFR* and *HER2* mutations by afatinib in NSCLC is more effective when these mutations are truncal dominant mutations (≥ 50%), as opposed to non-dominant (≥ 5 to < 50%) or low-frequency mutations (< 5%) [79]. DARWIN II (NCT02314481) is an exploratory phase II study examining the role of intratumor heterogeneity and predicted neo-antigens on the anti-tumor activity of anti-PDL1 immunotherapy [80]. Relationship between intratumor heterogeneity and cfDNA/CTCs will be explored, which may develop tools for patient selection and monitoring to be examined in future studies. Despite these studies are still in infancy, such endeavors might potentially refine treatment strategies to improve patient outcomes in the near future.

## Conclusions

**Fig. 2:**
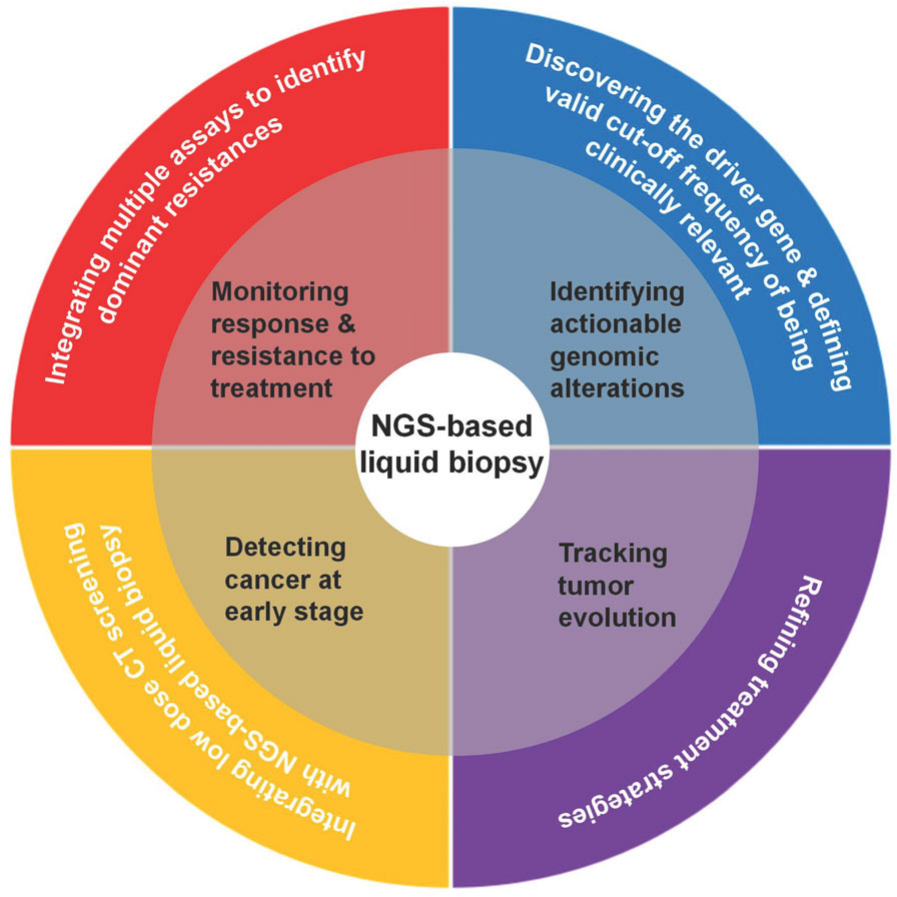
Current applications and future development of NGS-based liquid biopsy in lung cancer

The integration of NGS and liquid biopsy might complement the gold standard tissue testing and thrive to be a promising candidate of genomic profiling in NSCLC. NGS-based ctDNA assay might be applied in identifying actionable genomic alterations, dynamically monitoring response and resistance to targeted agents, prescreening early-stage lung cancer, and tracking spatiotemporal evolution of lung cancer (Fig. 2). However, challenges remain such as difficulties in distinguishing clinical meaningful driver genomic alterations, defining valid cut-off frequency of being clinically relevant, obstacles in identifying dominant resistance mechanisms, when to take action in case of positive ctDNA screening results in early-stage NSCLC, and cost-effectiveness of tacking tumor evolution. Further studies are warranted to overcome these challenges to define the clinical utility of NGS-based liquid biopsy.

## Abbreviations

ALK: Anaplastic lymphoma kinase
ARMS: Amplification Refractory Mutation System
CTCs: Circulating tumor cells
ctDNA: Circulating tumor DNA
EGFR: Epidermal growth factor receptor
HER2: Human epidermal growth factor receptor-2
HR: Hazard ratio
ICGC: The International Cancer Genome Consortium
MAF: Mutant allele fraction
MET: Mesenchymal–epithelial transition
MTL: Molecular tumor load
NGS: Next-generation sequencing
NSCLC: Non-small cell lung cancer
OS: Overall survival
PCR: Polymerase chain reaction
PPV: Positive predictive value
RECIST: Response Evaluation Criteria in Solid Tumors
RET: RET proto-oncogene
ROS1: c-ros oncogene 1 receptor
TCGA: The Cancer Genome Atlas
tDNA: Tissue DNA
